# Snoezelen, structured reminiscence therapy and 10-minutes activation in long term care residents with dementia (WISDE): study protocol of a cluster randomized controlled trial

**DOI:** 10.1186/1471-2318-10-5

**Published:** 2010-01-31

**Authors:** Almuth Berg, Katharina Sadowski, Melanie Beyrodt, Stephanie Hanns, Markus Zimmermann, Gero Langer, Christiane Becker, Christine Lautenschläger, Johann Behrens

**Affiliations:** 1 Institute for Health Care and Nursing Studies, Medical Faculty, Martin-Luther-University Halle-Wittenberg, Germany; 2 Department of Medical Psychology, University of Leipzig, Germany; 3 Institute of Public Health and Nursing Science, University of Bremen, Germany; 4 Institute of Medical Epidemiology, Biostatistics, and Informatics, Medical Faculty, Martin-Luther-University Halle-Wittenberg, Germany

## Abstract

**Background:**

People with dementia are often inapproachable due to symptoms of their illness. Therefore nurses should establish relationships with dementia patients via their remaining resources and facilitate communication. In order to achieve this, different targeted non-pharmacological interventions are recommended and practiced. However there is no sufficient evidence about the efficacy of most of these interventions. A number of publications highlight the urgent need for methodological sound studies so that more robust conclusions may be drawn.

**Methods/Design:**

The trial is designed as a cluster randomized controlled trial with 20 nursing homes in Saxony and Saxony-Anhalt (Germany) as the units of randomization. Nursing homes will be randomly allocated into 4 study groups consisting of 5 clusters and 90 residents: snoezelen, structured reminiscence therapy, 10-minutes activation or unstructured verbal communication (control group). The purpose is to determine whether the interventions are effective to reduce apathy in long-term care residents with dementia (N = 360) as the main outcome measure. Assessments will be done at baseline, 3, 6 and 12 months after beginning of the interventions.

**Discussion:**

This trial will particularly contribute to the evidence on efficacy of non-pharmacological interventions in dementia care.

**Trial Registration:**

ClinicalTrials.gov NCT00653731

## Background

Dementia is one of the most frequent and serious diseases occurring in people of higher age. There are currently more than 6 million people with dementia in the European Union with the prevalence of the disease increasing in the next decades [1].

People with dementia are often inapproachable due to symptoms of their illness. Besides cognitive disorders, primarily the non-cognitive symptoms of dementia, such as apathy, anxiety, depression, and challenging behavior, complicate the course of the disease and impede communication. Therefore nurses should establish relationships with dementia patients via their remaining resources and facilitate communication. Emotions, memories, important characteristics of the patients' biography, as well as perceptions on the somatic and emotional level can be used as resources in order to approach persons with dementia by using different pathways, even if communicative relationships are seemingly impeded.

In order to achieve this, different conceptualized non-pharmacological interventions are recommended in practice guidelines and practiced [2,3]. However, there is no sufficient evidence about the efficacy of several of these interventions, such as snoezelen and reminiscence therapy [4-7]. A number of publications highlight the urgent need for methodological sound studies so that more robust conclusions may be drawn [8,9].

The purpose of this study is to determine whether the interventions of snoezelen/multisensory stimulation, structured reminiscence therapy, and 10-minutes activation [10] are effective to reduce apathy in long term care residents with dementia.

## Methods

### Study design

A cluster randomized controlled trial with nursing homes as clusters is designed to verify the effects of 3 individual interventions: structured reminiscence therapy, 10-minutes activation and snoezelen. In order to preclude that the effects arise only due to the intensified social contacts, analogous individual contacts are implemented in the control group via unstructured verbal communication.

It is hypothesized that the interventions will have a positive impact on apathy in long term care residents with dementia in comparison to the control group. The primary outcome measures apathy after 12 months.

### Inclusion of clusters

A cluster is defined either as a nursing home by itself or a working ward of a large nursing home. None of the clusters may share facilities or staff.

10 nursing homes in the areas of Leipzig, Saxony, and Halle, Saxony-Anhalt (Germany) respectively, will be recruited that have implemented none of the study interventions in routine care prior to the study.

### Recruitment of individual participants

The identification and complete inclusion of participants must be done before cluster randomization to avoid foreknowledge of allocation and to reduce selection bias and empty randomized clusters [11,12].

Information and instruction of the participants will be done personally and in written form by trained nursing home staff, the consent to participate [13] will be obtained in writing from the residents. In case guardians are appointed, they will be informed about the possibility of taking part in the trial and will be asked for a participation of their charge in the trial.

In the total 20 nursing homes all residents fulfilling the inclusion criteria (suffering from dementia, informed consent in person or by legal guardian, age: ≥ 55 years) will be assessed using the *Mini Mental State Examination - MMSE *[14] or the *MMSE version for vision impairment - MMblind *[15] by the external investigators. Exclusion criteria are Korsakoff's syndrome or cognitive impairments from other causes than dementia.

Then from each cluster 18 participating residents with a cut-off value MMSE ≤ 24 or MMblind ≤ 17 [15] will be included using computer-generated random number tables.

### Cluster randomization

The 20 included nursing homes will be randomized into 4 study groups consisting of 5 clusters each, stratified according to province:

▪ structured reminiscence therapy (experimental group 1),

▪ 10-minutes activation (experimental group 2),

▪ snoezelen (experimental group 3),

▪ unstructured verbal communication (control group).

An individual randomization on the resident level does not seem possible out of methodical and practical reasons. A clear distinction between individual interventions on the resident level cannot be assumed in the training of different nurses in different interventions within one institution. Therefore, to avoid contamination and to achieve a feasible approach a cluster randomization design is chosen.

For concealed allocation of clusters the randomization list will be computer-generated by the Institute of Medical Epidemiology, Biostatistics, and Informatics, Halle/Saale, which is blind to the identity of the nursing homes and the included residents, after all baseline measurements.

**Figure 1:**
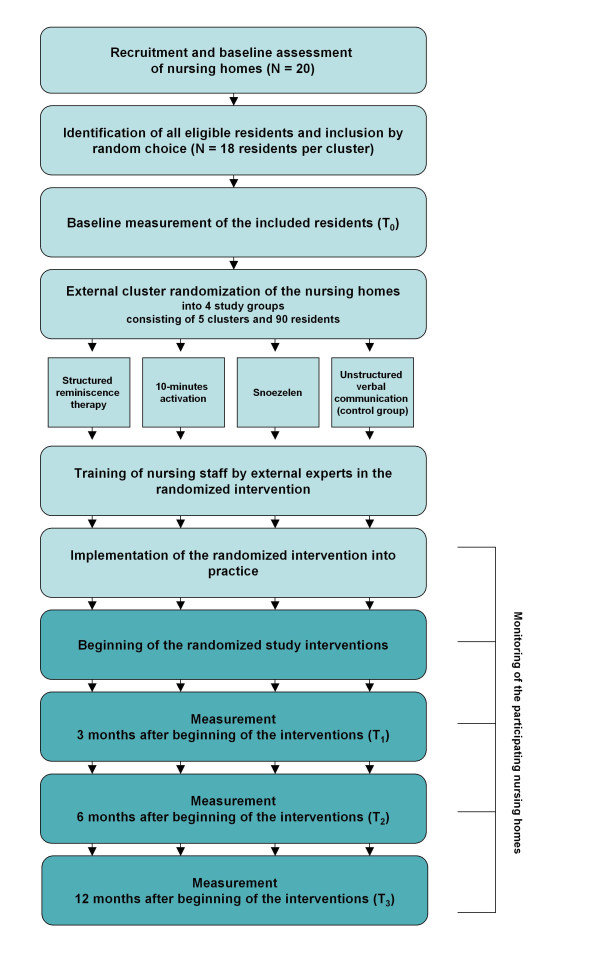
**Study design**.

The summary of the study design is presented in Figure 1.

### Study procedure

In each of the 20 included nursing home clusters 6 registered nurses will be selected and trained in the intervention their cluster will be randomized to. Nurses in the experimental groups receive training by external experts. Training takes place in three-day courses outside the facility. Instead of three-day training, nurses in the control group clusters will receive a one-day instruction on performing conversation-based individual contacts relating to everyday life.

After the training, the interventions in each of the participating nursing homes will be monitored by the investigators in order to evaluate the training effects. This is to secure that the interventions are carried out consistently and on the same level.

The interventions in the experimental groups will be carried out once or twice a week as one-to-one sessions by the trained nursing staff, over a period of 20 or 10 minutes over 12 months. This period was selected according to the intervention type and regards the feasibility in the nursing homes considering the time and personal resources.

In order to examine the possibility that effects can be observed solely due to intensified social contacts, the control group is designed as sham intervention on the basis of 20-minutes individual contacts once a week offering topical conversations.

Nursing staff will have to fill in a continuous documentation form about the topics and characteristics of every intervention session for every participating resident, including the case and reason of possible non-conduction. This documentation is intended to support the resident related intervention strategy and to enable a detailed data analysis about realized interventions.

All intervention facts are summarized in Table 1.

**Table 1:** Scheme of the study groups: study intervention program

Experimental group 1 (N = 5 nursing homes): Structured reminiscence therapy	
Education	3 days; 6 caregivers per nursing home
Intervention	• one-to-one session as an individual nursing intervention
	• once a week for 20 minutes
	• 3 residents per caregiver
Procedure	The aim of reminiscence is to stimulate memory and mood of demented people in the context of resident's life history, if applicable with the aid of sensory stimuli, e.g. photographs.
	
**Experimental group 2 (N = 5 nursing homes): 10-minutes activation**	
Education	3 days; 6 caregivers per nursing home
Intervention	• one-to-one session as an individual nursing intervention
	• twice a week for 10 minutes
	• 3 residents per caregiver
Procedure	The aim is to stimulate memory and all senses (visual, auditory, tactile and so on) by the use of different stimuli related to the resident and physical activation.
	

**Experimental group 3 (N = 5 nursing homes): Snoezelen**	
Education	3 days; 6 caregivers per nursing home
Intervention	• one-to-one session as an individual nursing intervention
	• once a week for 20 minutes
	• 3 residents per caregiver
Procedure	The aim is to improve the well-being of demented people by positive stimulation of their senses (visual, auditory, tactile, olfactory and gustatory stimulation) with different materials in a multisensory stimulation room or in the resident's room with a mobile snoezelen unit.
	

**Control Group (N = 5 nursing homes): Unstructured verbal communication**	
Instruction	1 day; 6 caregivers per nursing home
Active comparator	• one-to-one session as an individual nursing intervention
	• once a week for 20 minutes
	• 3 residents per caregiver
Procedure	The participants in the control group receive individual contacts with the same intensity by verbal conversation about everyday life without specific strategies.

### Measures

**Figure 2:**
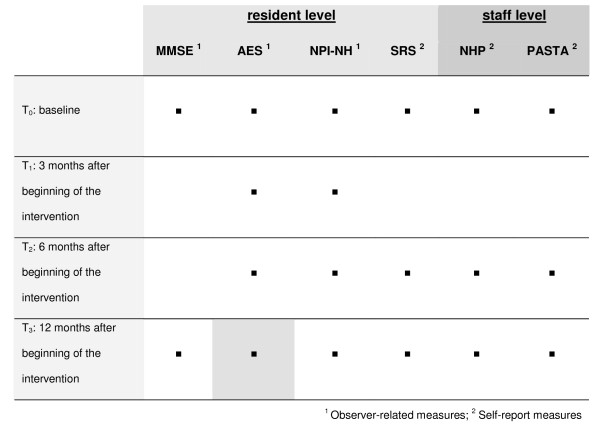
**Points of measurement and outcome measures**.

Evaluation of the interventions will be done on resident and staff level. Four points of measurement are scheduled in the trial: baseline assessment and measurements after 3, 6, and 12 months after intervention begin (see Figure 2).

The primary outcome measure is apathy after 12 months using the German version of the *Apathy Evaluation Scale - AES *[16,17]. Data collection will be done by interviews of the nursing staff.

Secondary outcome measures on the resident level are behavioral disturbances, isolated aggressive events and well-being.

Behavioral disturbances will be measured using the *Neuropsychiatric Inventory (Nursing Home) - NPI-NH *[18]. The instrument is a caregiver-based rating scale to evaluate 12 neuropsychiatric symptoms in patients with dementia: delusions, hallucinations, agitation, depression, anxiety, apathy, irritability, euphoria, disinhibition, aberrant motor behavior, night-time behavior disturbances, and appetite and eating abnormalities. Isolated aggressive events will be continuously documented by the staff members using the *Staff Observation Aggression Scale-Revised - SOAS-R *[19]. This instrument is to monitor the frequency, nature, and severity of aggressive incidents. Well-being will be measured using a Likert-type *Smiley Face Rating Scale *- *SRS *range from 1 to 4 marks by the residents.

Socio-demographic data like age, gender, and treatment data will be assessed and documented thoroughly in a Case Report Form, including the German versions of the *Clinical Dementia Rating *- *CDR *[20,21] and the *Care Dependency Scale: Pflegeabhängigkeitsskala *- *PAS *[22,23]. As a progressive course of disease can be assumed, a final assessment to the most important treatment data (i.e., MMSE, CDR, PAS) will be done for a better interpretation of results.

As further secondary outcome measures on staff level the health-related quality of life using the *Nottingham Health Profile *- *NHP *[24,25] as well as the evaluation of the subjective work pressure with subscales of the *Potentialanalyse stationärer Altenpflege - PASTA *[26] will be measured on the basis of a self-evaluation. It can be assumed that due to the increase of successful communication and the reduction of challenging behavior in residents with dementia, physical and mental strains of caregivers will be reduced and their health-related quality of life will increase or the strain caused by their job's tasks will be reduced.

### Sample size calculation

It is assumed that people with mild to moderate dementia have a mean apathy score *AES *of 45 [16]. To detect a mean reduction of the apathy score from 45 to 35 points (23%) with standard deviation (SD) 12 points between one intervention and one control group we need 13 residents in 10 nursing homes. The power of 80% for a cluster randomized t-test with a significance level alpha = 0.016 (0.05/3) is obtained with assumed intra-cluster correlation coefficient of ICCC = 0.05. The number of nursing homes increases to 20 (4 groups consisting of 5 nursing homes each) as there are three intervention groups and one control group and all intervention groups are tested against the same control group. Considering a mortality rate of 40%, the number of required study participants in each nursing home increases to 18 residents with dementia and thus to a total sample size of N = 360 residents (20 nursing homes * 18 residents).

### Blinding

In accordance to the mode of the interventions the residents and field researchers are not blinded to group allocation. A blinded statistician not involved in the trial process will be responsible for final analysis.

### Drop-outs

Drop-outs will be documented thoroughly and included in the data analysis until the point of drop-out. Reasons for loss to follow-up and protocol deviation will be reported and analyzed both at the level of the cluster (cluster withdrawal or loss to follow-up) and the individual (resident withdrawal or loss to follow-up) [12].

### Data analysis

All analyses are based on the intention-to-treat principle, which means that each cluster and the individuals (residents) are evaluated according to their randomly assigned intervention.

Metric variables will be reported using means and standard error of measurement. For nominal or ordinal variables this will be done by reporting rates with declaration of the 95% confidence interval. The changes of the primary outcome apathy score *AES *over time and additional data taken at the other measurement points are compared between the intervention and the control group and are treated as changes in proportions or means and their standard errors, according to the nature of the variables. Hypotheses tests concerning time effects, intervention effects, and an interaction time × intervention effect are carried out.

Correlated data which occurs if the residents are nested in the same cluster can be taken into account by applying fixed and random effects models (mixed models).

All results concerning the effects of intervention are primarily evaluated on individual level.

### Protection of data privacy

We will create a pseudonym for all participating nursing homes and residents to collect and analyze the trial data. Key lists will be stored separately from the trial data and erased after final data analysis. Data will be analyzed in a way that no conclusions can be drawn to individual participants or nursing homes. Trial data is stored in lockable cabinets in lockable rooms.

### Quality assessment

An accompanying monitoring by the investigators will assure the quality of the realized interventions and the collected data.

The trial is part of the Nursing Research Network "Mitte-Süd". A report system is established within the network. Annual quality reports have to be prepared for the German Federal Ministry of Education and Research as the funding organization.

### Publication policy

We plan to publish the trial results in a peer-reviewed, international, Medline-listed journal, independent of study results. All results will be reported within context to this study protocol and to the CONSORT statement extended to cluster randomized trials [27].

### Ethical considerations

The study protocol is approved by the ethics committee of the Medical Faculty, Martin-Luther-University Halle-Wittenberg, Germany.

Direct and indirect questioning of residents with dementia requires a particularly careful approach. Participation in the trial is voluntary and requires the consent of the residents and/or their legal guardians. Trial participants and/or their guardians will be receiving detailed information about the aims and the contents of the trial and will be referred to the application of the Federal Data Protection Act. The study will be carried out in compliance with the Helsinki Declaration.

## Discussion

Different nursing methods in the area of dementia are partly adopted in the practice with great euphoria but often without scientific evidence. Experiences show that there is therefore considerable need to identify adequate nursing methods in caring for people with dementia that are evidence-based and effective.

This trial will particularly contribute to the evidence on efficacy of non-pharmacological interventions in dementia care.

## Competing interests

The authors declare that they have no competing interests.

## Authors' contributions

KS, GL, SH, MZ, CB, and JB were responsible for identifying the research question and the general study design. AB, KS, and MB developed the study protocol. CL planned the statistical analysis, carried out the sample size calculation and was responsible as biometric counselor. AB was responsible for drafting this paper.

All authors commented on the paper drafts and read and approved the final manuscript.

## Pre-publication history

The pre-publication history for this paper can be accessed here:

http://www.biomedcentral.com/1471-2318/10/5/prepub
